# The influence of rotational thromboelastometry (ROTEM) on operating room and intensive care transfusion practices in major trauma bleeding: a prospective cohort study with historical control

**DOI:** 10.1186/s13741-025-00562-4

**Published:** 2025-07-21

**Authors:** Natalia Kozera, Marek Wełna, Waldemar Goździk

**Affiliations:** Clinical Department of Anesthesiology and Intensive Therapy, Wroclaw Medical University, Wroclaw, Poland

**Keywords:** ROTEM, Rotational thromboelastometry, Major trauma, Hemorrhage

## Abstract

**Background:**

Despite advances in treatment, hemorrhage remains one of the leading causes of early death in trauma. Rapid, personalized treatment of coagulopathy in this population should therefore be a priority. The introduction of viscoelastic hemostatic assays may improve transfusion strategies.

**Methods:**

This prospective observational study aimed to compare the efficacy of a ROTEM-guided hemostatic treatment protocol for trauma patients with a historical control group who had received conventional coagulation testing. The study included adults with multiple trauma requiring transfusion (≥ 1 unit of RBC within 12 h). The aim was to compare transfusion requirements in the operating room, on the 1st and 2nd ICU days, the rate of massive transfusion, and the overall outcome. The data obtained were stored in a database and analyzed using Statistica™ 13.3 (Stat Soft Polska). A *p*-value < 0.05 was considered significant. Study was registered retrospectively at researchregistry.com (RR10995).

**Results:**

A total of 78 patients were compared. The number of RBC units transfused in the OR and on the 1st ICU day decreased significantly after implementation of the ROTEM treatment protocol (*p* = 0.01, *p* = 0.04). Fewer patients in the study group required RBC transfusion on the 1st and 2nd ICU days (*p* = 0.01, *p* = 0.003), as well as the number of patients requiring FFP transfusion in all examined periods of time (*p* = 0.02, *p* = 0.006, *p* = 0.01). While FFP use per patient in the OR and on the 1st ICU day was lower, it was not statistically significant. Fibrinogen substitution in the OR remained similar, but more patients from the study group received it on the 1st ICU day (13 vs. 5, *p* = 0.04). The need for other blood products and coagulation factors remained unchanged. MT incidence decreased significantly in the first 24 h (*p* = 0.02), while 30-day mortality remained unchanged.

**Conclusions:**

The introduction of the ROTEM- guided hemostatic treatment protocol in trauma resulted in a changes in transfusion requirements and a reduction in the incidence of MT. ROTEM can be a useful clinical tool in the rapid and targeted management of bleeding trauma patients.

**Trial registration:**

Researchregistry.com (RR10995).

**Supplementary Information:**

The online version contains supplementary material available at 10.1186/s13741-025-00562-4.

## Introduction

According to the World Health Organization, major injury represents the most common cause of mortality among people aged 5 to 44 in high-income countries (Krug et al. 2000). Despite progress in treatment, exsanguination remains one of the primary causes of early trauma-related death (up to 40%) (Callcut et al. 2019). Trauma-induced coagulopathy (TIC), marked by fibrinolysis dysregulation, fibrinogen depletion, platelet dysfunction, and impaired thrombin generation, is associated with higher mortality (Moore et al. 2021). As hypofibrinogenemia plays a central role in severe bleeding (McQuilten et al. 2017), fibrinogen maintenance is crucial for reducing blood loss and transfusion needs. (Innerhofer et al. 2021; Innerhofer et al. 2017 Jun 1; Ziegler et al. 2021). However, the efficacy of FFP in treating coagulation disorders, particularly hypofibrinogenemia, is widely questioned (Khan et al. 2015; Collins et al. 2014; Rossaint et al. 2023; Görlinger and Saner 2015).

The latest European guidelines recommend the use of repeated conventional coagulation (CCA) and/or viscoelastic hemostatic assays (VHA) to guide hemostatic treatment (Rossaint et al. 2023). However, CCA are considered inadequate in the acute phase of TIC mostly due to the time required for results (Haas et al. 2015; Davenport et al. 2011; DeBot et al. 2024 Sep). Additionally, assessing blood clot quality using CCA alone is challenging, as they are stopped early, detecting only ~ 5% of thrombin generation (Sidonio et al. 2023). In the early stages of bleeding, Hb and CCA parameters may remain within normal ranges, potentially masking the severity of bleeding (Davenport et al. 2011). By contrast, VHA (ROTEM and TEG) evaluate the full clotting process—from fibrin cross-linking to clot breakdown. They deliver bedside results, enabling targeted treatment and providing valuable insights into fibrinogen function and fibrin polymerization (Innerhofer et al. 2017). Studies show VHA can reduce transfusion needs, particularly for FFP, and help identify patients at risk of massive transfusion (MT), potentially preventing this from happening. Thus, VHA use may lead to reduced morbidity, mortality, and treatment costs (Nardi et al. 2015; Stein et al. 2017; Gonzalez et al. 2016; Campbell et al. 2021; Schöchl et al. 2011a; Gorlinger et al. 2012).

In light of the presented evidence, we conducted a prospective cohort study with a historical control to compare the effectiveness of a ROTEM-guided hemostatic treatment protocol versus standard approach based on CCA in the trauma setting. We hypothesized that implementing the ROTEM-guided protocol would reduce the use of transfused blood products in the early phase of trauma (in the OR-Operating Room and during the first two days in the ICU-Intensive Care Unit). Particular attention was given to the demand for FFP transfusions. Our secondary hypothesis was that this approach would impact 30-day mortality, ICU length of stay, and the number of mechanical ventilation-free and vasopressor-free days.

## Materials and methods

### Study settings and design

A prospective cohort study with a historical control was performed at the Clinical Department of Anesthesiology and Intensive Therapy, University Hospital in Wroclaw. The study was conducted after obtaining approval from the Bioethics Committee at the Wroclaw Medical University, approval number KB-610/2016 dated 14.12.2016, extension approval number KB-685/2017 dated 02.11.2017. During the course of the study, the ROTEM-guided hemostatic treatment protocol was the local standard of care for patients at risk for TIC and at risk of massive post-traumatic bleeding. Informed consent was waived due to the observational nature of the study. Nevertheless, as soon as their general condition became stable, patients in the study group were informed and asked to provide written informed consent. In case of death, the next of kin were contacted.

### Study population

Patients over 18 years old with multiple body injuries (≥ 2 body areas) requiring blood product transfusion within 12 h after injury (≥ 1 RBC unit) were enrolled in the study. The exclusion criteria were as follows: age under 18 years, cardiac arrest before admission to the OR, pregnancy, prior anticoagulant and/or antiplatelet therapy.

The study group included patients treated between 01 January 2017 and 31 December 2019 according to the ROTEM guided hemostatic treatment protocol. The historical control group consisted of patients treated from 01 January 2015 until 31 December 2016 using CCA.

### Data acquisition

**Fig. 1:**
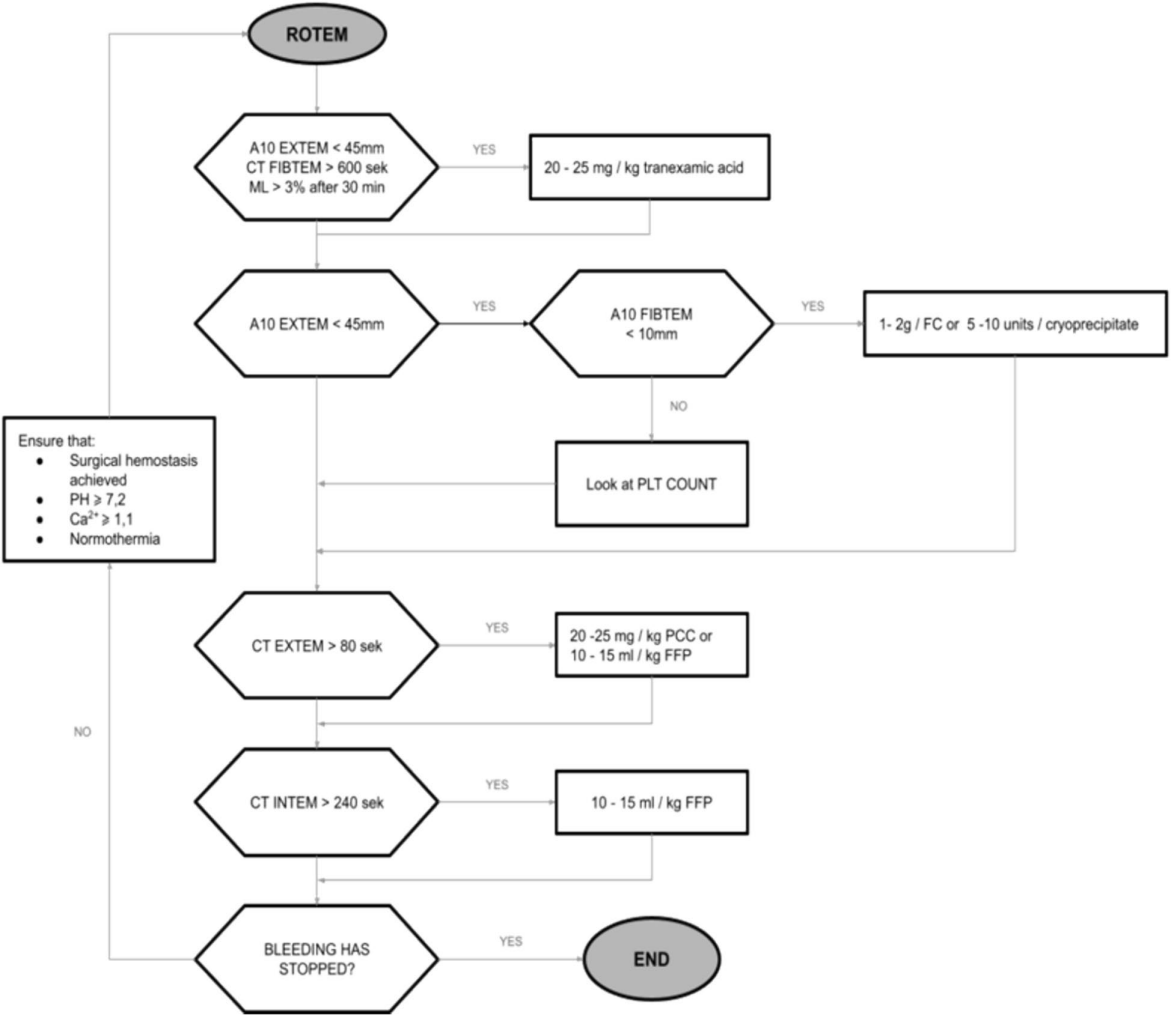
Implemented ROTEM guided hemostatic treatment protocol (ROTEM®-Rotational Thromboelastometry, CT—clotting time, A10—clot amplitude 10 min after CT, ML—maximum lysis, FC—fibrinogen concentrate, PLT—platelets, PCC–prothrombin complex concentrate, FFP–fresh frozen plasma) (Gorlinger et al. 2016)

Since 1 January 2017, patients with multiple body injuries who were treated according to the ROTEM guided hemostatic treatment protocol (Fig. 1) and met the inclusion criteria were enrolled in the study. The observation period started with the activation of the presented protocol (in the OR) and ended after the first 48 h of ICU stay. The obtained data were stored in an electronic database and subjected to analysis.

The control group was established through a process of medical record review, using data sourced from the hospital electronic database. The electronic documentation of patients admitted to our hospital after multiple trauma who met the established inclusion and exclusion criteria was identified and subjected to analysis. Between 2015 and 2016, 42 patients were identified. Four patients were ultimately excluded from the statistical analysis due to the incomplete documentation or lack of full medical history. Subsequently, 38 patients were included in the study as the control group. Records created prior to 2015 were excluded from the analysis due to the potential for incomplete data (prior to 2015, medical records were mainly kept in paper form).

### Sample size

The primary hypothesis of the study was that implementing the ROTEM algorithm would reduce blood product usage in the OR and during the first 2 days of ICU treatment, particularly FFP usage. The power and sample size were calculated using the ClinCalc calculator. We established that a total sample size of 54 patients would have 80% power to detect a minimum 50% reduction in FFP between groups with 95% confidence (Nardi et al. 2015; Gorlinger et al. 2012). Based on this information (and the annual historical number of patients meeting inclusion and exclusion criteria) 40 trauma patients treated according to the ROTEM algorithm were planned to be enrolled.

### The overall characteristics of both management strategies

Since 1 January 2017, the ROTEM algorithm (Fig. 1) has become the local standard of care for trauma patients at risk of trauma-induced coagulopathy and post-traumatic bleeding.

After enrollment in the study, arterial blood was collected for ROTEM analysis, blood count, and blood gas analysis. The ROTEM test was performed within 15 min using the ROTEM delta located in the OR. If laboratory or clinical signs of massive bleeding were present, the physician could order and initiate thawing of initial FFP units while awaiting ROTEM results, maintaining an FFP:RBC ratio of at least 1:2. As soon as possible, further hemostatic treatment was administered according to the ROTEM-guided hemostatic treatment protocol (Fig. 1). The protocol targeted three components of acute trauma coagulopathy: hyperfibrinolysis, reduced clot stability, and impaired thrombin generation. EXTEM, INTEM, and FIBTEM tests were performed to guide transfusion decisions based on the presented threshold values. In cases of hypofibrinogenemia (A10 EXTEM < 45 mm; A10 FIBTEM < 10 mm) or impaired thrombin generation (CT EXTEM > 80 s), the therapeutic agent recommended by the algorithm was selected by the treating physician based on the patient’s condition and clinical judgment (Fig. 1). The therapeutic intervention continued until hemostasis was achieved. If bleeding persisted despite adequate surgical hemostasis and adherence to all protocol conditions, the use of rFVIIa was considered. Additional ROTEM testing, along with laboratory coagulation assays, blood count, and arterial blood analysis, was performed upon ICU admission and after 24 h of ICU treatment, and whenever clinically indicated.

Prior to 2017, local standards for hemostatic care in multiple trauma relied on CCA with target thresholds detailed in Table 1.

**Table 1:** The overall characteristics of both management strategies for acute traumatic bleeding used

	2015–2016	2017–2019
Preconditions	rapid surgical hemostasis, pH > 7.2, BE > − 6, Ca2 + > 1.1, normothermia	rapid surgical hemostasis, pH > 7.2, BE > − 6, Ca2 + > 1.1, normothermia
Fluid therapy	crystalloids, avoiding colloids	crystalloids, avoiding colloids
Maneuvers to reduce excessive bleeding	Permissive hypotension-SAP 80–90 mmHg (*MAP 80 mmHg), avoiding hypervolemia	Permissive hypotension-SAP 80–90 mmHg (* MAP 80 mmHg), avoiding hypervolemia
Hb	7–9 g/dl	7–9 g/dl
Tranexamic acid	1 g in 3 h after injury + additionally 1 g in cases of massive hemorrhage	1 g in 3 h after injury + additionally 20–25 mg/kg if A10 EXTEM < 45 mm, CT FIBTEM > 600 s, ML 30 min > 3%
Uncontrolled bleeding strategy	FFP: RBC ratio > = 1:2	FFP: RBC ratio > = 1:2
Coagulation suport	aPTT, PT < 1.5 × normal, INR < 1.5, fibrinogen level > 1.5 g/l, platelet count > 50,000/mm^3^ (*100,000/mm^3^)	ROTEM test values according to introduced protocol; platelet count > 50,000/mm^3^ (*100,000/mm^3^)

* thresholds for TBI, *BE* base excess, *SAP* systolic arterial pressure, *MAP* mean arterial pressure, *FFP* fresh frozen plasma, *RBC* red blood cells, *aPTT* activated partial thromboplastin time, *PT* prothrombin time

Both groups were managed according to the European guidelines valid during the study period (Rossaint et al. 2016; Spahn et al. 2013). Key parameters essential for proper blood clotting were maintained, including a pH greater than 7.2, BE above – 6 mmol/L, ionized calcium (Ca^2^⁺) levels exceeding 1.1 mmol/L, and normothermia. Hemoglobin was kept between 7 and 9 g/dL, and crystalloids were the primary fluid. Excessive bleeding was managed using strategies such as permissive hypotension and the avoidance of hypervolemia prior to surgical hemostasis. In severe cases, an empiric FFP:RBC ratio of at least 1:2 was used prior to coagulation test results. Table 1 summarizes and compares both management strategies for acute traumatic bleeding.

### Collected data

The demographic data, severity of injuries estimated on anatomical and pathophysiological trauma scales (Injury Severity Score—ISS, Revised Trauma Score—RTS, Glasgow Coma Scale—GCS, Acute Physiology and Chronic Health Evaluation II—APACHE II, SAP and Shock Index—SI), standard coagulation laboratory data with the symptoms of trauma induced coagulopathy (aPTT > 1.5 × normal and INR > 1.5) and the estimated risk of massive transfusion (Trauma Associated Severe Hemorrhage—TASH score) were considered. The mechanism of injury was also taken into consideration. The frequency of injuries associated with a high risk of massive bleeding was determined and compared. All of the above were estimated using data collected upon admission to the Emergency Department (ED). Additionally, the tissue perfusion parameters (pH, BE, and lactates) upon admission to the OR were also compared.

### Outcome

The primary endpoint was the requirement for blood products and coagulation factor concentrates in the OR and during the initial 48 h of ICU treatment (1st and 2nd day of ICU). Since cryoprecipitate and fibrinogen concentrate were used interchangeably in cases of reduced clot stability, the total number of patients who received these agents was additionally analyzed. The number of massive transfusions (MT) was analyzed. Secondary endpoints included 30-day mortality, ICU and total hospital length of stay, mechanical ventilation free days, and vasopressor-free days. In light of the markedly elevated frequency of TBI coexistence observed in the control group, a subsequent analysis of length of ICU stay was conducted, focusing on patients with and without TBI coexistence.

### Definitions

Massive transfusion (MT) was defined as the transfusion of 10 or more units of RBC within the first 24 h after trauma (total RBC transfused from hospital admission). For blood products and coagulation factor concentrates, the following units were used: 1 unit of RBC (red blood cells; approx. 250 ml), 1 unit of FFP (fresh frozen plasma; approx. 230–260 ml), 1 unit of PLT (multiple-donor platelets or platelets from apheresis—depending on availability), 1 unit of cryoprecipitate (approx. 20 ml), 1 g of FC (lyophilized fibrinogen concentrate), 1 UI of PCC (lyophilized four-factor prothrombin complex concentrate; UI refers to FIX), 1 mg of rFVIIa (recombinant activated factor VII).

### Statistical analysis

The data were stored in a database and analyzed using the Statistica™ 13.3 (Stat Soft Polska). The obtained data are presented as median values with IQ1–IQ3 or as percentages, depending on the presented variable. The distribution of the data was evaluated using the Shapiro–Wilk test. Continuous variables were compared using either the *U* Mann–Whitney test or Student’s *t*-test, depending on the underlying distribution. For categorical variables, the chi-square test with Yates correction, if required, was used. The risk ratio for massive transfusion was calculated using a 2 × 2 table. A *p*-value < 0.05 was considered significant.

## Results

### Patient characteristics

**Fig. 2:**
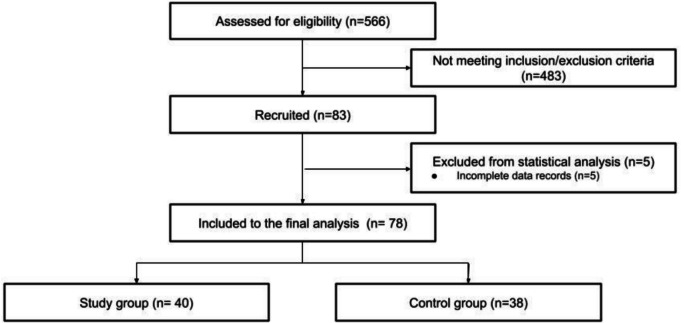
Flow diagram of the study

Of the 533 trauma patients admitted over the 5-year period, 83 met eligibility criteria and were recruited into the study. Five patients were excluded from statistical analysis due to missing data (Fig. 2).

A total of 78 records were analyzed, with both groups showing similar demographics, injury severity, risk of massive transfusion, and laboratory data (Table 2). The leading causes of injury were traffic accidents and falls. Blunt trauma was the predominant mechanism, occurring in 39 patients (97.5%) in the study group and 37 patients (97.3%) in the control group, while penetrating trauma was observed in 1 patient (2.5%) in the study group and 1 patient (2.7%) in the control group. Unstable pelvic fractures were observed in 15 patients in the study group and 13 in the control group (*p* = 0.76). Open or displaced femoral fractures were recorded in 16 patients from the study group and 14 from the control group (*p* = 0.77). Suspicion of free fluid in the peritoneal cavity (FAST/CT) was noted in 16 patients from the study group and 13 patients from the control group (*p* = 0.59). The control group had significantly lower GCS scores upon admission (*p* = 0.04) and a higher prevalence of TBI coexistence, with 18 cases compared to 9 in the study group (*p* = 0.02).

**Table 2:** The main characteristics of both examined groups

	Study group (*n* = 40)	Control group (*n* = 38)	*p* value
Demographic Age [med (Q1–Q3)] Sex—male/female [number (%)]	37.5 (27.5–50) 28 (70%)/12 (30%)	38 (28–57) 27 (71%)/11 (29%)	0.96 0.91
Severity of the injury ISS [med (Q1–Q3)] RTS [med (Q1–Q3)] APACHE II [med (Q1–Q3)] GCS [med (Q1–Q3)] TBI [%] SAP on admission to ED [med (Q1–Q3)] SI [med/Q1–Q3]	41 (34–43) 7 (4.4–7.8) 17 (11–22) 12 (5–15) 9 (22.5%) 90 (80–112.5) 1.1 (0.9–1.4)	34 (34–41) 4.5 (2.8–7.8) 15.5 (11–24) 5.5 (3–13) 18 (47.4%) 99.5 (80–110) 1.0 (0.8–1.5)	0.73 0.17 0.47 0.04 0.02 0.99 0.36
Estimated risk of massive transfusion TASH score [med (Q1-Q3)]	11 (8–17)	12 (6.5–18.5)	0.81
Laboratory data on ED admission Hb g/dl [med (Q1–Q3)] Plt thous/mm3 [med (Q1–Q3)] aPTT s [med (Q1–Q3)] INR [med (Q1–Q3)] INR > 1.5 and/or aPTT > 1.5 times [number (%)]	11 (9.5–12.85) 248.5 (173–299.5) 26.2 (22.9–29) 1.2 (1.1–1.3) 8 (20%)	10.7 (8.9–11.8) 222 (141–269) 31 (27.8–36.8) 1.3 (1.2–1.6) 10 (27%)	0.26 0.45 0.0004 0.02 0.3
Tissue perfusion parameters on OR admission BE [med (Q1–Q3)] Lac mmol/l [med (Q1–Q3)] pH [med (Q1–Q3)]	− 4.4 (− 8 to − 1.8) 3.4 (2.1–4.7) 7.28 (7.18–7.32)	− 4.8 (− 6.85 to − 2.7) 3.6 (2.7–4.85) 7.29 (7.24–7.33)	0.93 0.27 0.38

*ISS* Injury Severity Score, *RTS* Revised Trauma Score, *APACHE II* Acute Physiology and Chronic Health Evaluation II, *GCS* Glasgow Coma Scale, *TBI* Trauma Brain Injury, *SAP* Systolic Arterial Pressure, *SI* Shock Index, *TASH* Trauma Associate Severe Hemorrhage, *Hb* hemoglobin, *Plt* platelets, *aPTT* activated partial thromboplastin time, *INR* International Normalized Ratio, *BE* base excess, *Lac* lactates

Three patients from the study group and two from the control group were admitted directly to the ICU without requiring urgent surgical intervention. Comorbidities were present in 4 study group patients (epilepsy, schizophrenia, chronic pancreatitis, hypertension) and in 3 control group patients (type 1 diabetes, hypothyroidism, atherosclerosis).

### Outcome

Following the implementation of a ROTEM-guided hemostatic treatment protocol, no statistically significant difference in 30-day mortality rates was observed between the groups (7 vs. 7; *p* = 0.91). The main causes of death were: TBI (study group: 1 vs. control group: 2), hemorrhage (study group: 1 vs. control group: 3), sepsis (study group: 3 vs. control group: 0), and Multi-Organ Dysfunction Syndrome (MODS) (study group: 2 vs. control group: 2).

The median length of the ICU stay was shorter in the study group (9 vs. 14.5 days, respectively; *p* = 0.02). There was no significant difference in the median total length of hospital stay between the two groups (22 vs. 22 days, respectively; *p* = 0.6). In the subgroup with TBI coexistence, the median length of ICU stay was as follows: study group = 12.5 days, control group = 29.5 days (*p* = 0.03). In the subgroup without TBI coexistence, the median length of ICU stay was: study group = 8.5 days and control group = 9 days (*p* = 0.6). The median ventilator-free days did not differ between the groups (4 vs 4 days, respectively; *p* = 0.66). A significant improvement was observed in the median number of vasopressor-free days in the study group (5 vs 11 days, respectively; *p* = 0.01).

### Comparative blood product transfusion analysis

RBC and FFP transfusions were also performed during early trauma treatment in the ED, before the transfer to the OR. RBC transfusions were given to 19 patients from the study group and 13 from the control group (med = 2, IQR = 2 vs. med = 2, IQR = 1 respectively; *p* = 0.34). FFP was administered to 5 patients in the study group (med = 1, IQR = 0) and 1 patient in the control group (med = 2, IQR = 0); *p* = 1.0. No other blood-derived products were transfused in the ED.

Following the introduction of the ROTEM-guided hemostatic protocol, a significant reduction in RBC units transfused per patient was observed. The number of patients requiring RBC transfusion during the first two ICU days was also significantly lower. While FFP use per patient in the OR and on the 1 st ICU day was lower, the difference was not statistically significant. However, the number of patients needing FFP transfusion declined across all time intervals. PLT transfusion needs remained unchanged.

Fibrinogen concentrate dosage and the number of patients receiving it were comparable between groups, as well as cryoprecipitate requirements. The number of patients requiring fibrinogen substitution (with either fibrinogen concentrate and/or cryoprecipitate) in the OR remained comparable between groups (study group 11; control group 7; *p* = 0.45). However, a significantly higher number of patients in the study group received fibrinogen substitution on the first day in the ICU (study group 13; control group 5; *p* = 0.04).

PCC dosage per patient was similar between groups, with only one patient from the study group receiving it. Recombinant activated factor VII (rFVIIa) was administered intraoperatively to a single patient in the control group at a total dose of 8 mg; no other patients received rFVIIa during the study period.

The tranexamic acid dosage per patient was comparable between the groups. However, a significantly higher number of patients in the study group received it in the OR.

A comparative analysis of blood product transfusion requirements is provided in Table 3. The number of patients who received each blood product and factor concentrate is outlined in Table 4.

**Table 3:** A comparative analysis of blood product transfusion demands presented for both examined groups

	Study group (*n* = 40)	Control group (*n* = 38)	*p* value
RBC unit [med (Q1–Q3)] Operating room 1 st day of ICU stay 2nd day of ICU stay	2 (2–4) 2 (1–2) 2 (2–2)	4 (2–8) 2 (2–4) 2 (1–2)	0.01 0.04 0.7
FFP unit [med (Q1–Q3)] Operating room 1 st day of ICU stay 2nd day of ICU stay	2 (2–3) 2 (2–2) 1 (1–8)	3 (2–5) 3 (2–5) 2 (1–3)	0.06 0.07 0.87
PLT unit [med (Q1–Q3)] Operating room 1 st day of ICU stay 2nd day of ICU stay	10 (10–10) 10 (6–11) 5.5 (5–6)	10 (9.5–10) 10 (10–17) 10 (9–12)	0.56 0.44 0.14
Cryoprecipitate unit [med (Q1–Q3)] Operating room 1 st day of ICU stay 2nd day of ICU stay	8 (4.5–10) 7.5 (2–10) 10 (2–10)	4.5 (3–6) – –	0.46 – –
FC g [med (Q1–Q3)] Operating room 1 st day of ICU stay 2nd day of ICU stay	1 (1–2) 1.5 (1–2) 2 (2–2)	1 (1–2) 2 (2–4) –	0.76 0.13 –
PCC UI [med (Q1–Q3)] Operating room 1 st day of ICU stay 2nd day of ICU stay	– 600 (600–600) 600 (600–600)	1000 (500–1000) 1000 (1000–1000) –	– 1.0 –
Tranexamic acid g [med (Q1–Q3)] Operating room 1 st day of ICU stay 2nd day of ICU stay	1 (1–1) 1 (1–1.5) –	1 (1–1) 1 (1–1) –	0.7 1.0 –

*RBC* red blood cells, *FFP* fresh frozen plasma, *PLT* platelets, *FC* fibrinogen concentrate, *PCC* prothrombin complex concentrate, *rFVIIa* recombined activated factor VII

**Table 4:** The proportion (%) of patients who received each blood product and factor concentrate across all examined periods

	Study group	Control group	*p* value
RBC [number of patients (%)] Operating room 1 st day of ICU stay 2nd day of ICU stay	36 (97.3%) 17 (42.5%) 14 (35.0%)	35 (97.2%) 27 (71.1%) 26 (68.4%)	0.98 0.01 0.003
FFP [number of patients (%)] Operating room 1 st day of ICU stay 2nd day of ICU stay	18 (48.6%) 12 (30.0%) 3 (7.5%)	27 (75.0%) 23 (60.5%) 11 (28.9%)	0.02 0.006 0.01
PLT [number of patients (%)] Operating room 1 st day of ICU stay 2nd day of ICU stay	3 (8.1%) 4 (10.0%) 2 (5.0%)	4 (11.1%) 7 (18.4%) 3 (7.9%)	0.96 0.45 0.95
Cryoprecipitate [number of patients (%)] Operating room 1 st day of ICU stay 2nd day of ICU stay	4 (10.8%) 5 (12.5%) 1 (2.5%)	2 (5.6%) 0 (0.0%) 0 (0.0%)	0.69 0.07 0.32
FC [number of patients (%)] Operating room 1 st day of ICU stay 2nd day of ICU stay	7 (18.9%) 8 (20.0%) 1 (2.5%)	5 (13.9%) 5 (13.2%) 0 (0.0%)	0.79 0.81 0.32
PCC [number of patients (%)] Operating room 1 st day of ICU stay 2nd day of ICU stay	0 (0.0%) 1 (2.5%) 1 (2.5%)	3 (8.3%) 5 (13.2%) 0 (0.0%)	0.22 0.18 0.32
Tranexamic acid [number of patients (%)] Operating room 1 st day of ICU stay 2nd day of ICU stay	20 (55.5%) 8 (20.0%) 0 (0.0%)	8 (22.8%) 4 (10.5%) 0 (0.0%)	0.01 0.39 –

*RBC* red blood cells, *FFP* fresh frozen plasma, *PLT* platelets, *FC* fibrinogen concentrate, *PCC* prothrombin complex concentrate

### Massive transfusion rate

In the study group, 3 patients (7.5%) required MT within the 24-h post-injury period, compared to 11 patients (28.9%) in the control group (*p* = 0.02). The relative risk (RR) for MT was 0.25 (95% CI 0.07 to 0.86), with a number needed to treat (NNT) of 4.66 (95% CI 2.65 to 19.66). The *p*-value was 0.02. The mean predicted risk of a MT, calculated using the TASH score, was 11% for the study group, while the actual incidence was 7.5%. Among patients who received MT, 8 died: 2 in the study group and 6 in the control group (*p* = 0.7).

## Discussion

The objective of this study was to assess the impact of a ROTEM-guided hemostatic treatment protocol (Fig. 1) in trauma settings, with a particular focus on its influence on blood consumption in the OR and during the first 48 h in the ICU. Both examined groups had comparable demographics, injury mechanisms, and injury severity. However, the control group had a significantly lower GCS score, due to a higher incidence of TBI coexistence.

We hypothesized that implementing the ROTEM algorithm (Fig. 1) would reduce blood product use, especially fresh frozen plasma (FFP). Similar retrospective studies have reported a 65–94% decrease in FFP utilization following the introduction of ROTEM-guided protocols (Nardi et al. 2015; Stein et al. 2017; Gorlinger et al. 2012). Although a decreasing trend in FFP use was observed both in the OR and on the 1 st ICU day—with reductions of 35% and 41.5%, respectively—the number of FFP units transfused per patient did not change significantly. However, the proportion of patients receiving any FFP transfusion was significantly lower across all evaluated time intervals. Bainbridge et al. reported similar findings, noting a reduction in the proportion of patients receiving FFP, but with an accompanying increase in the number of patients receiving cryoprecipitate (Bainbridge et al. 2021). This effect may reflect ROTEM’s advantages, including rapid turnaround and comprehensive coagulation assessment, enabling early transition from empirical therapy to goal-directed management using more appropriate agents than FFP when needed. Gratz et al. reported that ROTEM results were available earlier than CCA (33 min vs. 71 min), with abnormal CCA results in 5 of 32 patients compared to 21 of 32 in the ROTEM group (Gratz et al. 2019). Although the ITACTIC study showed no impact on the primary outcome (alive and free from MT at 24 h) or FFP requirements, the frequency of hemostatic interventions was higher in the VHA group compared to the CCA group (67% vs. 36%, respectively), with significantly increased fibrinogen use (Baksaas-Aasen et al. 2021). Similarly, Riehl et al. observed more frequent hemostatic treatment (69.5% vs. 41.7%) and increased fibrinogen substitution (54.4% vs. 29.1%) following ROTEM implementation (Riehl et al. 2022).

In light of the presented evidence and considering the importance of hypofibrinogenemia in trauma-induced coagulopathy (McQuilten et al. 2017), we anticipated increased fibrinogen replacement after the ROTEM-guided hemostatic protocol (Fig. 1) implementation. However, both the fibrinogen concentrate dose and the proportion of patients receiving it remained unchanged, consistent with Stein et al. (Stein et al. 2017) The observed effect may partly result from the use of cryoprecipitate as a substitute for fibrinogen concentrate during the study period. Both the CRYOSTAT and FEISTY trials demonstrated that cryoprecipitate may be an effective option for fibrinogen replacement (Curry et al. 2015; Winearls et al. 2017). Many studies, including the ITACTIC trial, aggregate fibrinogen supplementation data without distinguishing cryoprecipitate from concentrate, requiring assumptions about fibrinogen content per cryoprecipitate unit (0.15–0.25 g), which can vary. To avoid this, we presented the doses of both products separately. However, we also calculated the proportion of patients who received fibrinogen supplementation, either via fibrinogen concentrate and/or cryoprecipitate. While the proportion of patients requiring fibrinogen in the OR was similar between groups (11 vs. 7, *p* = 0.45), significantly more patients in the study group received fibrinogen on the first ICU day (13 vs. 5, *p* = 0.04).

It is worth noting that PCC was not administered at all in the OR in the study group during our study, and only one patient required its use within the first 48 h in the ICU. Similarly, Görlinger et al. reported a 36% reduction in PCC use after ROTEM implementation, but with an eightfold increase in fibrinogen substitution (Gorlinger et al. 2012). Campbell et al. found similar results (Campbell et al. 2021). Since severe hypofibrinogenemia affects both CCA and VHA tests, including also CT and CFT (Gratz et al. 2020; Hofmann et al. 2025; Ponschab et al. 2015), it should always be considered when interpreting ROTEM results and before deciding whether to administer PCC. Notably, PCC increases thrombin generation for several days after a single dose, and this effect may not correspond with CCA or VHA results (Schöchl et al. 2014). The PRCOAG study showed that initial PCC administration in bleeding trauma patients does not reduce blood product use, but may significantly increase thromboembolic complications (Bouzat et al. 2023).

Hyperfibrinolysis is a key component of trauma-induced coagulopathy, and tranexamic acid is essential in trauma bleeding management (Rossaint et al. 2023). The implemented ROTEM-guided hemostatic algorithm took into account not only the percentage of clot lysis as a marker of hyperfibrinolysis but also a prolonged FIBTEM clotting time (CT > 600 s), indicating severely impaired or even absent clot formation, as well as reduced clot stability (A10 EXTEM < 45 mm). As a result, a significantly higher number of patients with severe coagulation abnormalities in the study group received tranexamic acid in the OR, although the total dose administered remained unchanged.

Our study demonstrated a significant reduction in red blood cell (RBC) transfusions in the OR and on the first ICU day after ROTEM-guided treatment introduction (Fig. 1). Large volumes of FFP can lead to fluid overload and hemodilution; therefore, early use of coagulation factor concentrates instead may have a beneficial impact also on RBC consumption (Khan et al. 2015). Schochl et al. demonstrated that quick ROTEM-guided administration of coagulation factor concentrates helped to completely avoid RBC transfusion in 29% of patients compared to those treated only with FFP (Schöchl et al. 2011b). However, several retrospective studies evaluating the impact of ROTEM on blood management in trauma, despite a reduction in the demand for FFP, did not confirm a corresponding decrease in RBC (Nardi et al. 2015; Stein et al. 2017; Campbell et al. 2021).

Following the introduction of a ROTEM-guided hemostatic protocol (Fig. 1), the frequency of MT significantly decreased (3 vs. 11 patients, respectively), despite the initial TASH score indicating a similar calculated risk of MT for both groups. The MT predicted risk for the study group was 11%, whereas the actual rate was 7.5%. These findings are consistent with those reported by Nardi et al. (33 vs. 16 patients, respectively) and Stein et al. (40 vs. 15 patients, respectively) (Nardi et al. 2015; Stein et al. 2017). The ITACTIC study did not confirm these observations, although high administration of blood products prior to randomization (9 patients received MT before enrollment) may have influenced the results (Baksaas-Aasen et al. 2021).

Multiple studies highlight VHA’s efficacy in identifying MT risk, modifying transfusion requirements, reducing MODS incidence, mortality, and costs in trauma (Nardi et al. 2015; Stein et al. 2017 Nov 1; Gonzalez et al. 2016; Campbell et al. 2021 Jun 1; Bainbridge et al. 2021 Oct 1; Riehl et al. 2022; Lammers et al. 2020; Salehi et al. 2023). Meta-analyses by Da Luz et al. and Veigas et al. support VHA’s value for early trauma-induced coagulopathy diagnosis and MT prediction, though benefits on mortality remain unclear (Veigas et al. 2016; Luz et al. 2014). Although the ROTEM-guided hemostatic treatment protocol (Fig. 1]) led to changes in blood management practices during our study, mortality rates remained comparable between the groups. The study group demonstrated a shorter duration of ICU stay and an increase in vasopressor-free days. However, these findings were likely influenced by a significantly higher incidence of traumatic brain injury (TBI) in the control group. After re-analysis excluding patients with TBI, the difference in ICU length of stay was no longer statistically significant. Schöchl et al. and Campbell et al. also found no evidence that ROTEM reduces ICU or hospital stay (Campbell et al. 2021). Interestingly, the subgroup with TBI treated according to the ROTEM-guided hemostatic protocol maintained a shorter treatment time in the ICU. Nevertheless, the limited number of patients in each subgroup restricts the ability to reach clear conclusions.

## Limitations

It is important to highlight several limitations of this study.

The type of study we conducted is prone to confounding factors and has a high risk of selection bias. Although multiple assumptions were made to minimize their impact, it is important to consider this when interpreting the results. We tried to address confounding factors, but we acknowledge that not all variables influencing injured patient therapy can be identified. Moreover, we do not have complete data on pre-hospital treatment (especially regarding the tranexamic acid use).

The study was conducted at a single center, limiting the sample size and potentially underpowering the ability to assess ROTEM’s impact on overall outcomes. While some changes in transfusion policy were observed, along with a decrease in MT incidence, these findings were inconsistent, and no statistical significance was reached, particularly concerning FFP. The likely explanation is the smaller-than-expected reduction in FFP demand after ROTEM introduction and the small sample size.

Additionally, the ROTEM-guided hemostatic treatment protocol was not used in the ED; it was first performed in the OR. Therefore, the analysis focuses on the type and amount of blood products administered in the OR and over the first 48 h of the ICU stay. The results, however, provide a brief comparison of ED transfusion data between both groups.

Retrospective data collection for the control group lacked standardized timing, preventing a comparative analysis of timeframes, particularly for lab results and transfusion decisions. Records prior to 2015 were excluded due to changes in our hospital’s record-keeping system (from paper to electronic), which increased the risk of incomplete data and errors. Additionally, evolving treatment methods over time could introduce confounding variables. Therefore, we limited the database search to the past 2 years.

The control group had a significantly higher GCS score and more patients with coexisting TBI, likely affecting outcomes like ICU stay and ventilation-free days. After excluding TBI patients, ICU stay was similar between groups, but the small subgroup sizes limit clinical conclusions.

## Conclusions

ROTEM is a valuable tool for managing coagulopathy in trauma patients, offering a more comprehensive assessment and potentially altering transfusion strategies. After implementing the ROTEM-guided hemostatic protocol, changes in the administration pattern of blood products and a lower MT rate were observed. However, due to the small sample size, the full impact of ROTEM on blood management and patient outcomes could not be fully assessed. Larger, prospective, randomized studies are needed to better understand ROTEM’s role in trauma hemorrhage management.

## Supplementary Information

Supplementary Material 1.

## Data Availability

No datasets were generated or analysed during the current study.

## Abbreviations

SAP: Systolic arterial pressure
MAP: Mean arterial pressure
ROTEM: Rotational thromboelastometry
TEG: Thromboelastography
VHA: Viscoelastic assays
CCA: Classic coagulation assays
OR: Operating room
ICU: Intensive care unit
MT: Massive transfusion
RBC: Red blood cells
FFP: Fresh frozen plasma
PLT: Platelets
FC: Fibrinogen concentrate
PCC: Prothrombin Complex Concentrate
rFVIIa: Recombinant factor VIIa
aPTT: Activated partial thromboplastin clotting time
PT: Prothrombin time
INR: International Normalized Ratio
Hb: Hemoglobin
BE: Base excess
Lac: Lactates
TBI: Traumatic brain injury
ISS: Severity Injury Score
RTS: Revised Trauma Score
GCS: Glasgow Coma Scale
APACHE II: Acute Physiology and Chronic Health Evaluation II"
SI: Shock Index
ED: Emergency Department
TASH: Trauma Associated Severe Hemorrhage
FAST: Focused Assessment with Sonography in Trauma
CT: Computed tomography
NNT: Number needed to treat
RR: Risk ratio
MODS: Multi-Organ Dysfunction Syndrome
