# Supplementary material for: The influence of rotational thromboelastometry (ROTEM) on operating room and intensive care transfusion practices in major trauma bleeding: a prospective cohort study with historical control
Source: Perioper Med (Lond). 2025 Jul 21;14:75. doi: 10.1186/s13741-025-00562-4 (PMC12281821; doi:10.1186/s13741-025-00562-4)
Supplement: Supplementary file 1 — Supplementary Material 1. [file 13741_2025_562_MOESM1_ESM.docx]

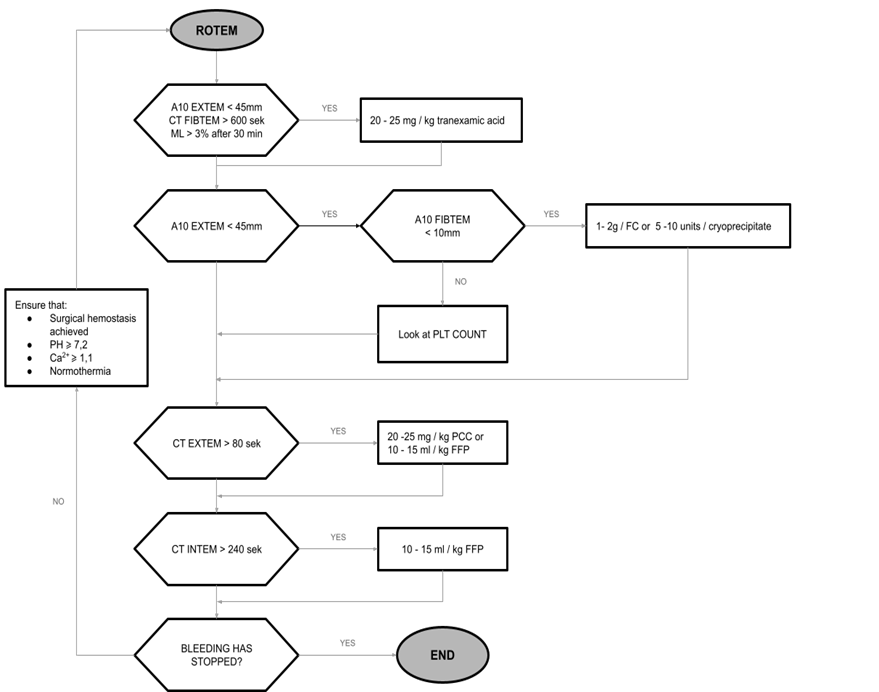


# **Figure 1.** Implemented ROTEM guided hemostatic treatment protocol (ROTEM® - Rotational Thromboelastometry, CT - clotting time, A10 - clot amplitude 10 min after CT, ML - maximum lysis, FC – Fibrinogen Concentrate, PLT – Platelets, PCC – Prothrombin Complex Concentrate, FFP – Fresh Frozen Plasma). (22)
